# Bacterial-microplastic interaction can affect disinfection efficiency of methods routinely used in the food industry

**DOI:** 10.3389/fnut.2026.1812387

**Published:** 2026-08-19

**Authors:** Tea Movsesijan, Natalie Gawin, Nina Gruber, Leon Ploszczanski, Christian Zafiu, Patrick Mikuni-Mester

**Affiliations:** 1 Unit of Food Microbiology, Centre for Food Science, Clinical Department for Farm Animals and Food System Transformation, University of Veterinary Medicine Vienna, Vienna, Austria; 2 Christian-Doppler Laboratory for Detection and Reduction of Dormant Bacteria, Centre for Food Science and Veterinary Public Health, University of Veterinary Medicine Vienna, Vienna, Austria; 3 Department of Material Sciences and Process Engineering, Institute of Physics and Materials Science, BOKU University, Vienna, Austria; 4 Department of Landscape, Water and Infrastructure, Institute of Waste Management and Circularity, BOKU University, Vienna, Austria

**Keywords:** chemical, disinfection, food, microplastics, pathogen, thermal, UV

## Abstract

High abundance of microplastics in the environment and its potential dangers have been an increasingly emerging topic in the last decade. Thus far, research on microplastics has focused mainly on water systems, sewage, and soil, where they have been described as additional surfaces and vectors for microorganism growth and transmission. In our study we hypothesized that microplastics play a role in aiding the survival of bacteria in clinical or food production environments by influencing effectiveness of routine disinfection. To test this hypothesis, two typical foodborne pathogens, *Listeria monocytogenes* and *Escherichia coli*, were exposed to different disinfection methods, physical (heat and ultraviolet (UV) radiation) and chemical (lactic acid and benzalkonium chloride), in presence of microplastics. Two experimental groups were tested and subsequently compared with the control group: bacteria incubated overnight in presence of microplastics, and bacteria mixed with microplastics shortly before exposure to disinfectant methods. Our study included two different types and sizes of microplastics: 1–5 μm thermoset amino-formaldehyde (TAFP) polymers and 425–500 μm polyethylene (PE). Disinfection efficiency was determined from the log₁₀ reduction in bacterial counts after 10, 30 or 60 min of exposure to physical and chemical disinfectants, and after 60 min of exposure to UV. Our results indicate that, while microplastics do not shield the bacteria from heat-shock, they can support their survival in presence of benzalkonium chloride and under the exposure of UV light. More specifically, TAFP appears to cancel out the effect of benzalkonium chloride by absorbing or reacting with it. On the other hand, both types of microplastics seem to create a physical barrier protecting bacteria against UV light. Our research points out the necessity in taking microplastics into account when assessing disinfection methods efficiency.

## 1 Introduction

Microplastics are defined as plastic particles of different shapes with sizes < 5 mm (1). These particles have different sources, being either produced in sizes < 5 mm or fragmented in the environment from larger plastic objects (2). Due to their omnipresence in the technosphere microplastics emissions to the environment are inevitable (2). This emerging pollutant is also suspected to cause environmental and human health risks directly by their size and shape and indirectly by oligomers and additives leaching out of plastics (3). Human exposure depends on the type of particle and its size, with inhalation and dietary intake being the major routes of uptake (4). Dietary intake represents an important route of intervention, with different sources from the primary production (5) to the food processing sector. Food processing includes mechanical processes and packaging with inevitable wear from plastic materials from machines and packages used in facilities (6). In addition, surfaces can be contaminated through airborne plastic particles (7). Recent studies indicate that seafood contamination often arises from contact materials rather than the species itself (8, 9). However, data on oral uptake and quantitative risk assessments, especially for complex, processed foods, remain limited and consequently only few mitigation strategies have been developed.

Sources of contamination are plastics used for food packaging, which were traditionally made from polypropylene (PP), polystyrene (PS), polyvinylchloride (PVC), polyethylene (PE), polyethylene terephthalate (PET), polyamide (PA), ethylen-vinyl alcohol copolymer (EVOH), expanded PS (EPS), expanded PP (EPP) and expanded PE (EPE) (10) and are more recently replaced by biodegradable alternatives, such as polylactic acid (PLA) (11). Moreover, systematic evidence suggests that food contact materials, often made from polymers represent an important and often unnoticed source of micro- and nanoplastics (MNP) contamination in food (12).

In addition to the potential direct health implications, MNPs can carry hazardous substances and pathogenic microorganisms, which are the main hazard in food processing facilities for human health (13). The reduction of pathogens is made by so-called HACCP (Hazard Analysis and Critical Control Points) that demands disinfection and testing to reduce potential hazards from pathogenic microorganisms in the food industry. However, when a co-contamination of pathogens co-occurs with microplastics, they can act as “hosts,” providing an exceptional environments for these microorganisms, the so-called plastisphere (14). Additionally, recent studies show that in water/wastewater disinfection microplastics can provide a protective function impeding disinfection procedures (15, 16). Moreover, microplastics may also act as vectors for pathogens and provide improved conditions for microbial spread within a food production facility (17), yet data on migration patterns, pathogen attachment, and interactions with food-associated chemistry, including cleaning and disinfection, are scarce (18, 19). Improved resistance of pathogens, either by physical and chemical protection by microplastics, or fostering of microbial diversification can help to bypass the currently implemented preventive methods for food safety. Therefore, microplastic pollution and its mitigation in food processing should be considered in the future.

To address the emerging aspect of the consequences of microplastic contaminations in the food production we focused on fundamental experiments to understand the disinfection efficiency of microplastic and microorganism contaminated surfaces relevant for the food industry. The underlying hypothesis is that microplastic contamination aids bacterial survival during standard disinfection procedures in food production and clinical settings. Therefore, the study was designed to investigate how MNP properties and environmental factors influence interactions with biotic agents and assess their role as vectors for pathogens and co-contaminants in food systems. Our focus lied on the evaluation of MNP effects on disinfection efficacy (thermal, chemical, UV), identifying underlying mechanisms such as adsorption or shielding, and exploring mitigation strategies.

## 2 Materials and methods

### 2.1 Microplastics used in this study

Two types of microplastics were tested in this series of experiments: 425–500 μm polyethylene (PE; density 1.015 g/cm^3^, green fluorescent, Cospehric CC, USA) and 1–5 μm thermoset amino formaldehyde polymer (TAFP; density 1.3 g/cm^3^, red fluorescent, Cospehric CC, USA). PE is extensively used for food packaging and food contact materials and one of the most abundant polymer from which microplastics are made (20). TAFP and derivatives of it is a polymeric nanomaterial material are used for coatings (21) and as adhesive for wood (22). The microplastics used in this study were not sterilized, however it was handled aseptically and was controlled for contamination.

Both MNPs were tested at a deliberately high concentration of 1% (w/v) to assess their general potential to interfere with different disinfection methods. This concentration was chosen for two reasons. First, for practical reasons: with the larger PE particles used, homogeneous suspensions could not be prepared at lower concentrations because only a few particles (30,639–8,816) are present per sample at 1% (w/v) and we wanted to test both MNPs at the same mass concentration. Second, consistent with standard disinfection efficacy testing, which employs very high bacterial loads, potential interfering substances are evaluated at elevated levels. For example, interference from organic contamination in surface disinfection is assessed under “clean” (0.03% w/v organic compound, OC) and “dirty” (0.6% w/v OC) conditions according to the VAH (Verbund für Angewandte Hygiene) guidelines.

### 2.2 Bacterial strains and growth conditions with MNPs

Overnight cultures of *E. coli* (MG 1655) and *L. monocytogenes* (EGDe) were inoculated in Tryptic soy broth with added yeast (TSB + Y, Biokar Diagnostics, Allonne, France), from single colonies growing on Tryptic soy agar with added yeast (TSA + Y, Biokar Diagnostics, Allonne, France) and incubated at 37 °C. For inactivation experiments two different experimental and one control condition were used, representing different MNP contamination scenarios.

**Figure 1:**
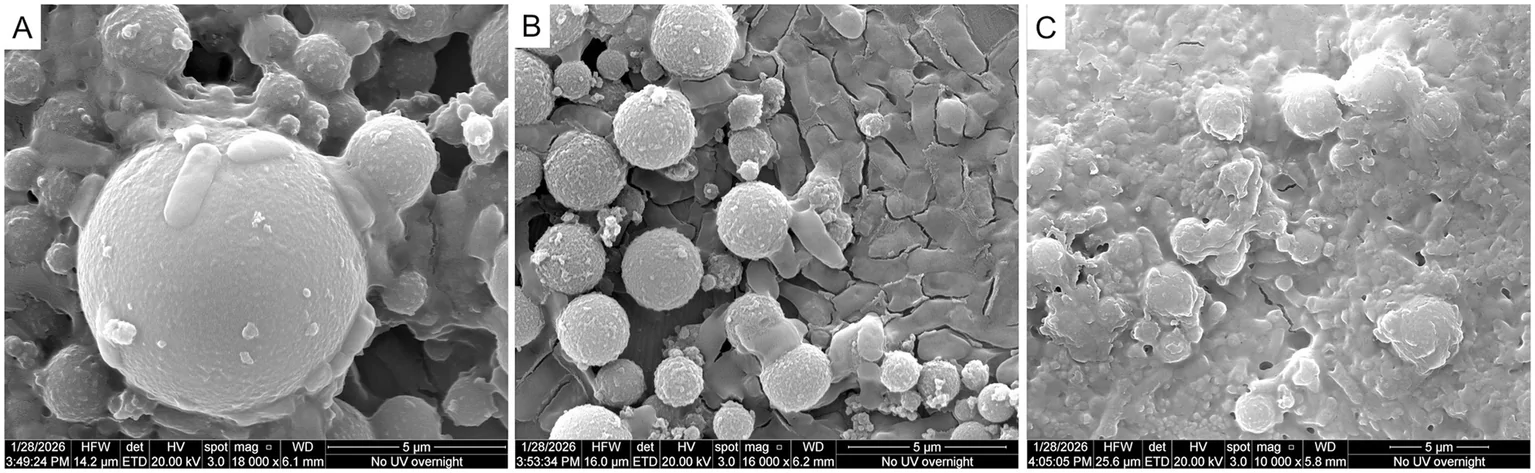
Bacteria and microplastic interaction. *Escherichia coli* (MG 1655) was tested in the presence of TAFP particles. Pictures **(A,B)** represent bacteria that were exposed to microplastics only briefly (group 2), while picture **(C)** shows bacteria incubated with microplastics overnight (group 1).

In the first condition (Group 1), overnight cultures were prepared from single colonies growing on TSA + Y, mixed with 10 mg/mL of the respective MNPs, incubated overnight at 37 °C and subsequently used for inactivation tests. This group represented conditions similar to those of a plastisphere. It was described in literature that bacteria are not only able to adhere to microplastics, but are also able to withstand high pressures and survive attached to the surface of the microplastics (23). As exemplary shown for *E. coli* and TAFP, the cells were able to form a biofilm covering both plastic particles and bacterial cells (Figure 1C).

In the second condition (Group 2), representing co-occurrence of MNPs and pathogens, overnight cultures from each bacterial species were used directly. We have chosen to test both of these groups in order to differentiate whether the biofilm formation around microplastics, or simply their presence aids the survival of bacteria. The optical density of the overnight culture was measured using a spectrophotometer, adjusted to the respective concentration for each experimental setup (see below), and subsequently mixed with 10 mg/mL of MNPs for inactivation tests. Bacterial cultures without any microplastics were used as control groups. As exemplary shown for *E. coli* and TAFP, the cells in group 2 could marginally bind to larger plastic particles (Figure 1A), while the majority was not directly bound (Figure 1B). Quantification of bacterial cell counts before and after the respective experimental condition was performed using the serial dilution method. In detail, cultures were serially diluted in 1x Phosphate-Buffered Saline (PBS), spot plated (10 μL per dilution) on TSA + Y and incubated at 37 °C. Colonies were counted after 20 h to obtain bacterial concentrations (log_10_(CFU/mL)).

### 2.3 Thermal inactivation

Thermal inactivation of all treatment groups was performed by incubating 500 μL of the respective samples at 60 °C, a temperature commonly used for inactivation of microorganisms (24). A 25 μL sample was taken after 10, 30 and 60 min of heat exposure, serially diluted in PBS and spot plated in the same way as previously described. All groups were tested in triplicates (*n* = 3).

### 2.4 Chemical inactivation

To test the efficiency of chemical disinfectants in the presence of MNPs, two disinfectants commonly used in the food production industry, benzalkonium chloride and lactic acid, were chosen. Chemical inactivation was performed with benzalkonium chloride (0.001% final concentration) and lactic acid (1.5% final concentration) using a similar experimental approach with minor differences. The concentrations used for these two substances were chosen to be at the level achieving disinfection (> 4 log_10_ reduction compared to the control), however low enough so that growth still remains detectable (Supplementary Figure 1). This allowed us to accurately quantify the log_10_ reduction through disinfection and correctly assess the effect of microplastics. Additionally, concentrations in this range have been reported in literature for testing of the inactivation abilities of these two chemicals (25, 26). In case of benzalkonium chloride, the optical density (600 nm) of the bacterial cultures was adjusted to OD_600_ = 0.22, followed by mixing 100 μL the MNP/bacteria with 1 mL of the disinfectant (Group 2). Aliquots (100 μL) of each sample were taken after 10, 30 and 60 min and centrifuged for 1 min at 13000 x g. The supernatant was aspirated, pellets were resuspended in 100 μL PBS and then serially diluted and spot plated as previously. The experiment was performed in biological triplicates (*n* = 3) for PE and with six biological replicates (*n* = 6) for TAFP. The number of replicates was increased for TAFP in order to ensure a more statistically relevant and robust sample size to evaluate the observed effect. Exposure to lactic acid was performed in a similar manner, however instead of adjusting the overnight culture to OD_600_ 0.22, undiluted overnight cultures of *L. monocytogenes* were used for the experiment. The experiment was performed in biological triplicates (*n* = 3) for all groups.

In order to test why benzalkonium chloride was ineffective at the tested concentration in presence of TAFP particles, the samples were exposed to benzalkonium chloride for 60 min, and subsequently filtered it using a 0.20 non-pyrogenic sterile μm filter (Filtropur S, Sarstedt AG & Co. KG, Germany). The filtered benzalkonium chloride was then used for a chemical inactivation of *E. coli*, as described. Bacterial culture samples were taken before exposure to benzalkonium chloride and after 60 min of exposure. The effect of filtered benzalkonium chloride was compared to *E. coli* Group 2 and *E. coli* without any microplastics. The experiment was performed in biological triplicates (*n* = 3).

### 2.5 Radiation inactivation

Mimicking a realistic scenario for surface decontamination, for radiation inactivation a built-in UV-light (254 nm) (27) within a biosafety cabinet was used (Thermo Scientific™ MSC-Advantage™ Class II Biological Safety Cabinet). For each experimental run, 100 μL aliquots of the 1:10 diluted respective samples were each dropped onto two sterile metal plates and dried at room temperature. After the metal plates were dried, one plate was exposed to UV irradiation UV light for 1 h while the other sample remained unexposed and served as a control. Afterwards, the plates were transferred to 15 mL tubes containing 12 glass beads (5 mm Glass beads, Carl Roth GmbH & Co. Kg, Karlsruhe, Germany) and 2 mL PBS. The tubes were vortexed for 3 min to remove the bacteria from the plates and subsequently spot plated in the same way as previously described. This method of bacterial isolation was adapted from (28). The experiment was performed in biological triplicates (*n* = 3) for all groups.

### 2.6 Scanning electron microscopy

For the scanning electron microscopy (SEM) investigations, a FEI Quanta 250 FEG (Thermo Fisher Scientific, Hillsboro, OR, United States) was used under high-vacuum conditions and a variable high tension from 2 to 20 kV. The micrographs were recorded with the Everhart–Thornley detector in secondary electron (SE) mode. The specimens were sputter-coated with a 10-nm-thin layer of gold in order to provide sufficient electrical conductivity.

### 2.7 Visualization and statistical analysis

All visualisation and statistical analysis were conducted in R Studio (v4.4.1). The statistical significance of the chemical inactivation results were conducted using the Wilcoxon Rank-Sum Test with Bonferroni adjustment, whereas the significance of the thermal inactivation results was assessed using the Kruskal–Wallis test, followed by Dunn’s post-hoc test with Bonferroni adjustment using the dunn.test R package (29). Plots were generated using ggplot2 (30), patchwork (31) and svglite (32).

## 3 Results and Discussion

In this study we aimed to determine whether the presence of microplastic can affect disinfection efficacy of methods routinely used in the food industry. For this purpose, we tested three disinfectant types: chemical (benzalkonium chloride and lactic acid), physical (heat) and radiation (UV light) in combination with two different types of microplastics differing in both composition and size (425–500 μm PE and 1–5 μm TAFP). The effects were tested on two of the most relevant foodborne pathogens, *E. coli* and *L. monocytogenes,* representing a gram-negative and gram-positive bacterial species.

### 3.1 Presence of microplastics does not reduce the efficiency of heat-based disinfection

**Figure 2:**
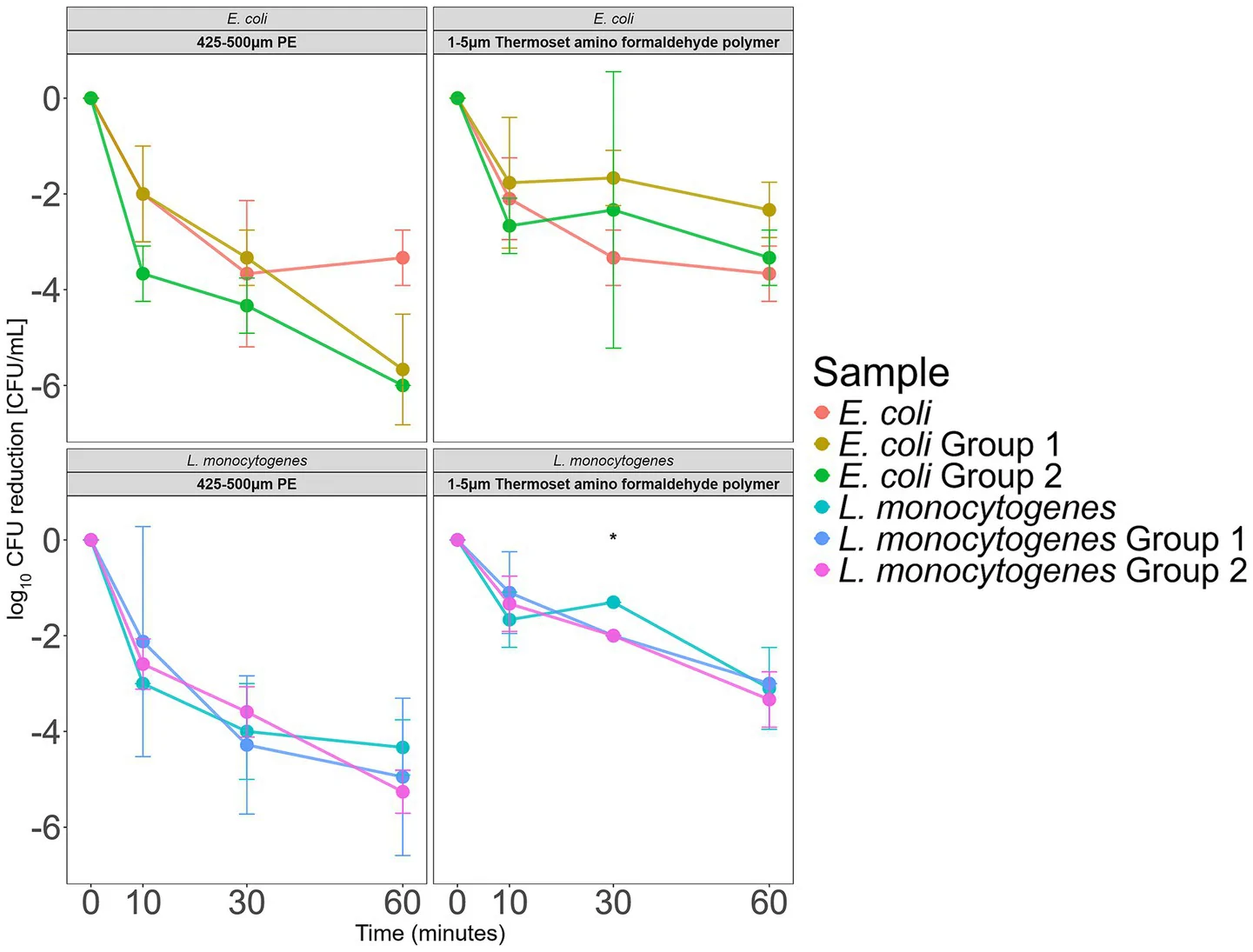
Effect of heat exposure on bacterial growth in the presence of microplastics. Two different species were used in the experiment: *Escherichia coli* (MG 1655) and *Listeria monocytogenes* (EGDe). Both species were tested alone and in the presence of PE or TAFP. Group 1 represents bacteria that were incubated with microplastics overnight, while Group 2 represents bacteria that were exposed to microplastics only briefly before the heat exposure. The dots represent mean log_10_ CFU reduction (CFU/mL) of the species after 0, 10, 30 and 60 min of exposure to 60 °C. Error bars indicate the standard deviation (*SD*). Sample sizes: *Escherichia coli* (*n* = 3 per condition), *Listeria monocytogenes* (*n* = 3 per condition). Statistically significant differences between the cultures were determined using Kruskal–Wallis test, followed by Dunn’s post-hoc test with Bonferroni adjustment (* *p* < 0.05, ** *p* < 0.01 and *** *p* < 0.001).

As expected, within 60 min of thermal inactivation at 60 °C, a 4 log_10_ reduction of viable cells was achieved for both pathogens in the control group (Figure 2). The results in the MNP conditions showed that their presence did not provide a survival advantage for *E. coli* and *L. monocytogenes* subjected to thermal inactivation.

For *E. coli,* results were variable, however similar inactivation kinetics could be observed for all groups. After 60 min of thermal inactivation both Group 1 and Group 2, as well as the control group, had a 2–6 log_10_ reduction, regardless of MNP used, (Figure 2; Supplementary Table 1). The results for *L. monocytogenes* mostly confirmed the results for *E. coli*, showing generally no visible effect of microplastics on survival in comparison to the bacterial control group (Figure 2; Supplementary Table 1). Only one significant difference (*p* = 0.014) was observed between the Group 1 and the control after 30 min of incubation at 60 °C. However, taking into consideration that there was no difference in inhibition after 10 min of heat exposure, and the low number of samples per group, we do not believe that this result indicates higher chances of survival in the presence of microplastics. Previous studies reported improved stability of viral particles in presence of MNPs, however for bacteria no such reports yet exist (33, 34). TAFP aids the survival of bacteria exposed to benzalkonium chloride.

### 3.2 Chemical inactivation

**Figure 3:**
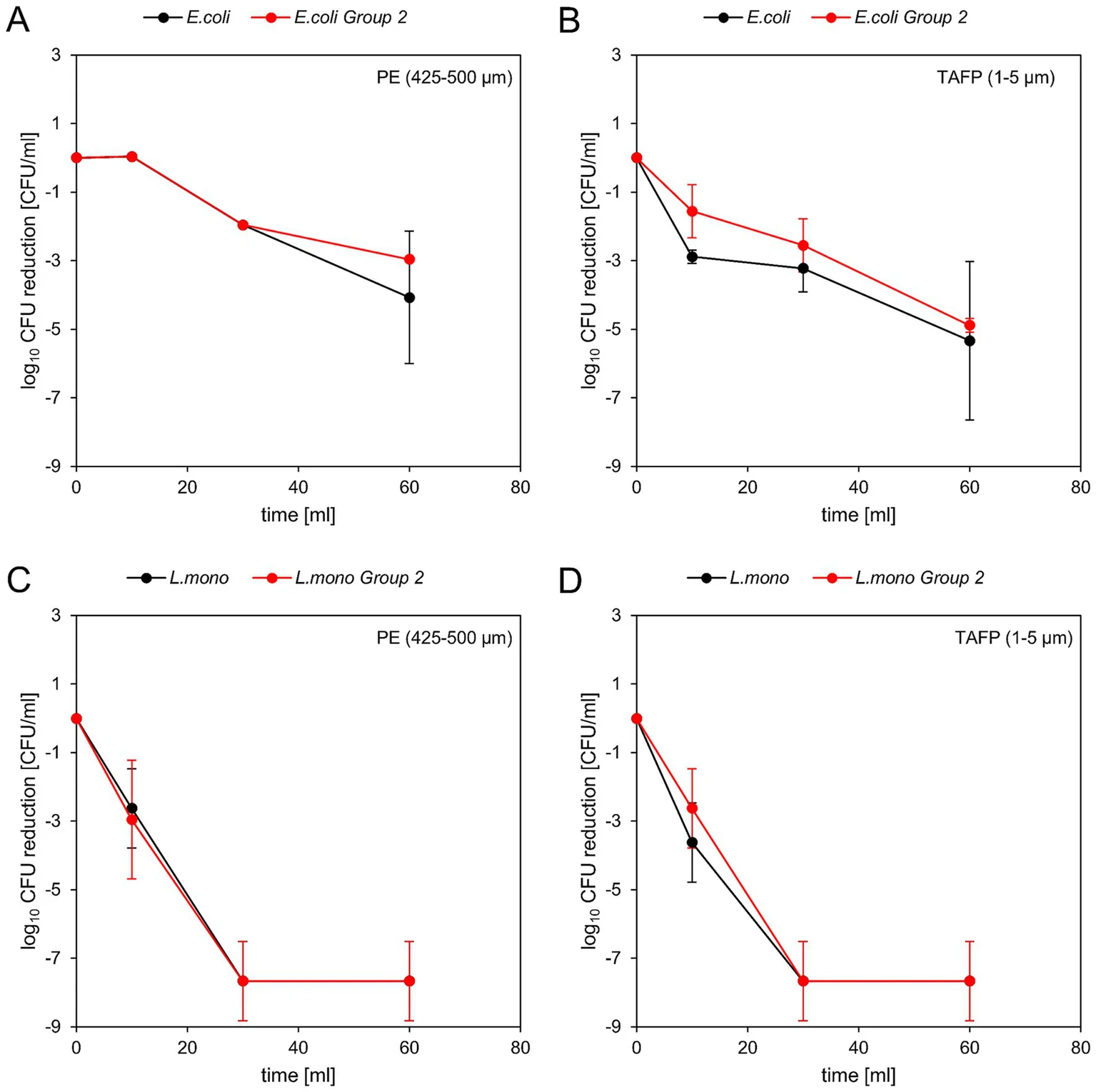
Effect of exposure to lactic acid on bacterial growth in the presence of microplastics. Bacteria were exposed to 1.5% lactic acid and were sampled after 0, 10, 30, and 60 min. Two different species were used in the experiment: **(A)** and **(B)** *Escherichia coli* (MG 1655); and **(C)** and **(D)** *Listeria monocytogenes* (EGDe). Both species were tested alone and in the presence of PE or TAFP. The dots represent mean log_10_ CFU reduction (CFU/mL) after subtraction of the initial bacterial concentration and the corresponding control concentration at each time point. Error bars indicate the standard deviation (SD). Sample sizes: *Escherichia coli* (*n* = 3 per condition), *Listeria monocytogenes* (*n* = 3 per condition). Statistically significant differences between the cultures were determined using Wilcoxon Rank-Sum Test with Bonferroni adjustment (*p < 0.05, ** *p* < 0.01, and ***p < 0.001).

To test a potential impact of MNP presence on chemical inactivation, two active compounds (benzalkonium chloride 0.001% and lactic acid 1.5%) of high relevance in the food industry were investigated. Similar to the results obtained for thermal inactivation, presence of either of the two MNP types did not affect antimicrobial efficiency of lactic acid, achieving a reduction of more than 4 log_10_ viable cells within 60 min for both pathogens (Figure 3; Supplementary Table 3).

**Figure 4:**
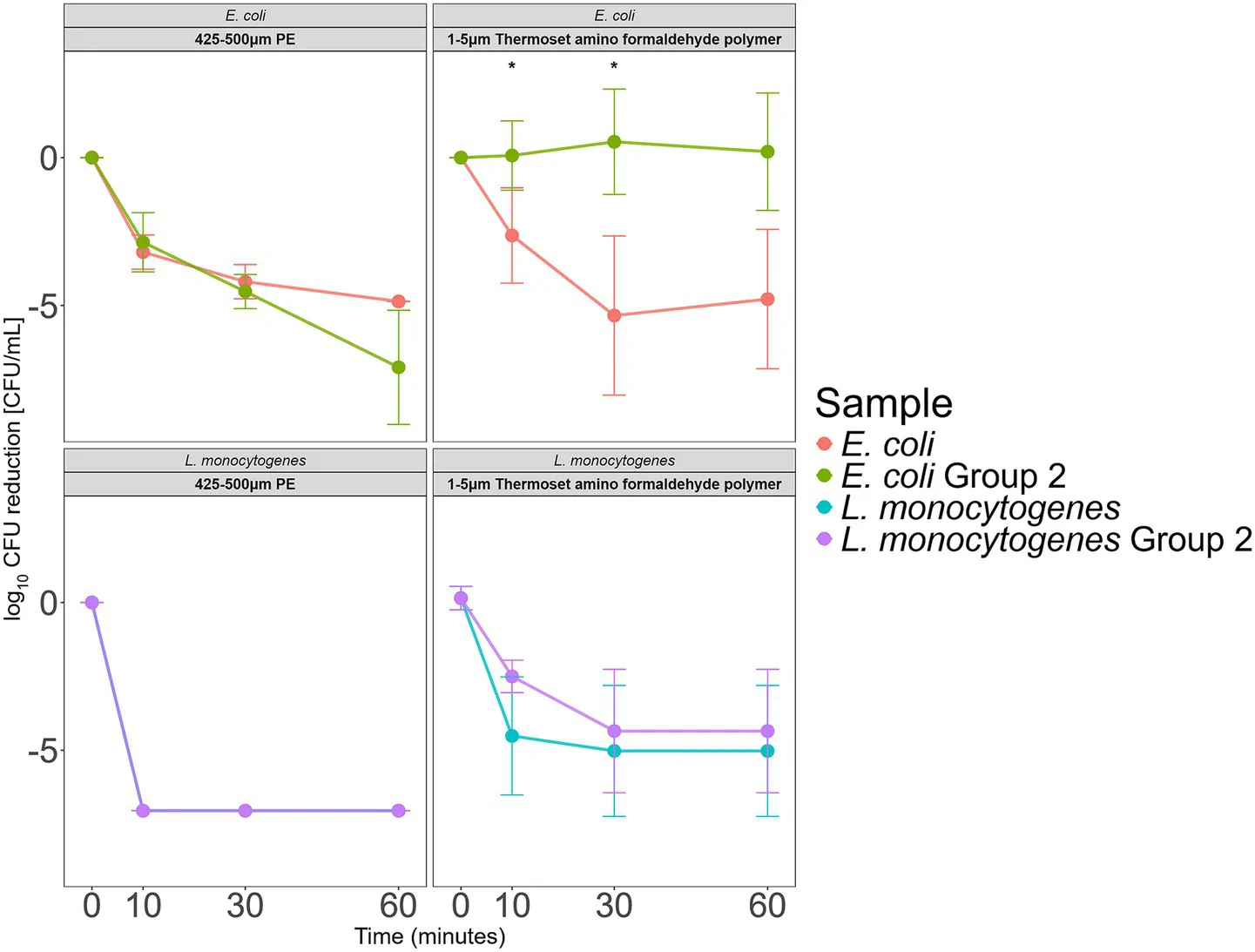
Effect of benzalkonium chloride on bacterial growth in the presence of microplastics. Bacteria were exposed to 0.001% benzalkonium chloride and were sampled after 0, 10, 30 and 60 min. Two different species were used in the experiment: *Escherichia coli* (MG 1655) and *Listeria monocytogenes* (EGDe). Both species were tested alone and in the presence of PE or TAFP. The dots represent mean log_10_ CFU reduction (CFU/mL) after subtraction of the initial bacterial concentration and the corresponding control concentration at each time point. Error bars indicate the standard deviation (*SD*). Sample sizes: *Escherichia coli* (PE: *n* = 3, TAFP: *n* = 6), *Listeria monocytogenes* (PE: *n* = 3, TAFP: *n* = 6). Statistically significant differences between the cultures were determined using Wilcoxon Rank-Sum Test with Bonferroni adjustment (* *p* < 0.05, ** *p* < 0.01, and *** *p* < 0.001).

For benzalkonium chloride, in case of *L. monocytogenes* again no effect of MNPs on its antimicrobial efficacy could be determined (Figure 4; Supplementary Table 2). In contrast, for *E. coli* a clear influence was found in the presence of one type of the MNPs. While PE microplastic did not appear to affect the efficiency of benzalkonium chloride, *E. coli* seemed to be less affected by benzalkonium chloride treatment in the presence of TAFP (Figure 4; Supplementary Table 2). This trend remained true throughout the entire 60 min exposure time, with significant differences between Group 2 and the bacterial control after 10 and 30 min of exposure (*p* = 0.04 at both time points). To test if the observed effect could be explained by a preferential binding of benzalkonium chloride to the MNPs, thus reducing the effective concentration of the disinfectant, we exposed benzalkonium chloride (0.001%) to TAFP for 60 min, filtered it to remove MNPs and subsequently used it for a chemical inactivation of *E. coli*. Incorporation of antimicrobials into plastics is normally achieved under high pressure (35), however a preferential interaction between the MNPs and the lipophilic benzalkonium chloride in comparison to the hydrophilic lactic acid could be possible. Our test showed that after MNP co-incubation, filtered benzalkonium chloride caused a low level of inactivation of *E. coli*, similar to the observed lack of chemical inactivation when TAFP was present in the chemical inactivation test (Supplementary Figure 2; Supplementary Table 4). These tests do not allow to reveal the mechanism behind the observations but indicate that benzalkonium chloride cannot perform in the presence of TAFP-MNP. In contrast to PE-MNPs, TAFP has a profound mesh-like structure, providing a high surface area for possible bacterial interactions and a different surface chemistry. Therefore, future studies should investigate a wider range of particle sizes and types from different plastics, as well as their surface properties and interaction with chemical disinfectants to elaborate on the exact mode-of-action. Additionally, another study (36) tested the toxicity of benzalkonium chloride towards *Daphnia magna* in presence of pristine and aged PE-MNPs, and a general absorption of benzalkonium chloride by PE-MNPs. They found that the length of the alkyl chains and the age and porosity of PE-MNPs played an important role in adsorption of benzalkonium chloride and subsequently it’s toxicity towards *D. magna.* While we did not observe any decrease in effectiveness of benzalkonium chloride in the presence of PE-MNPs, it is worth adding the perspective of age and wear of MNPs in future research.

### 3.3 PE and TAFP provide protection against UV light

In order to test the efficiency of UV light as a disinfection method in presence of MNPs, we exposed Group 1, Group 2 and bacterial controls to 60 min of UV-treatment and compared them to the individual controls from unexposed samples of the same three groups.

**Figure 5:**
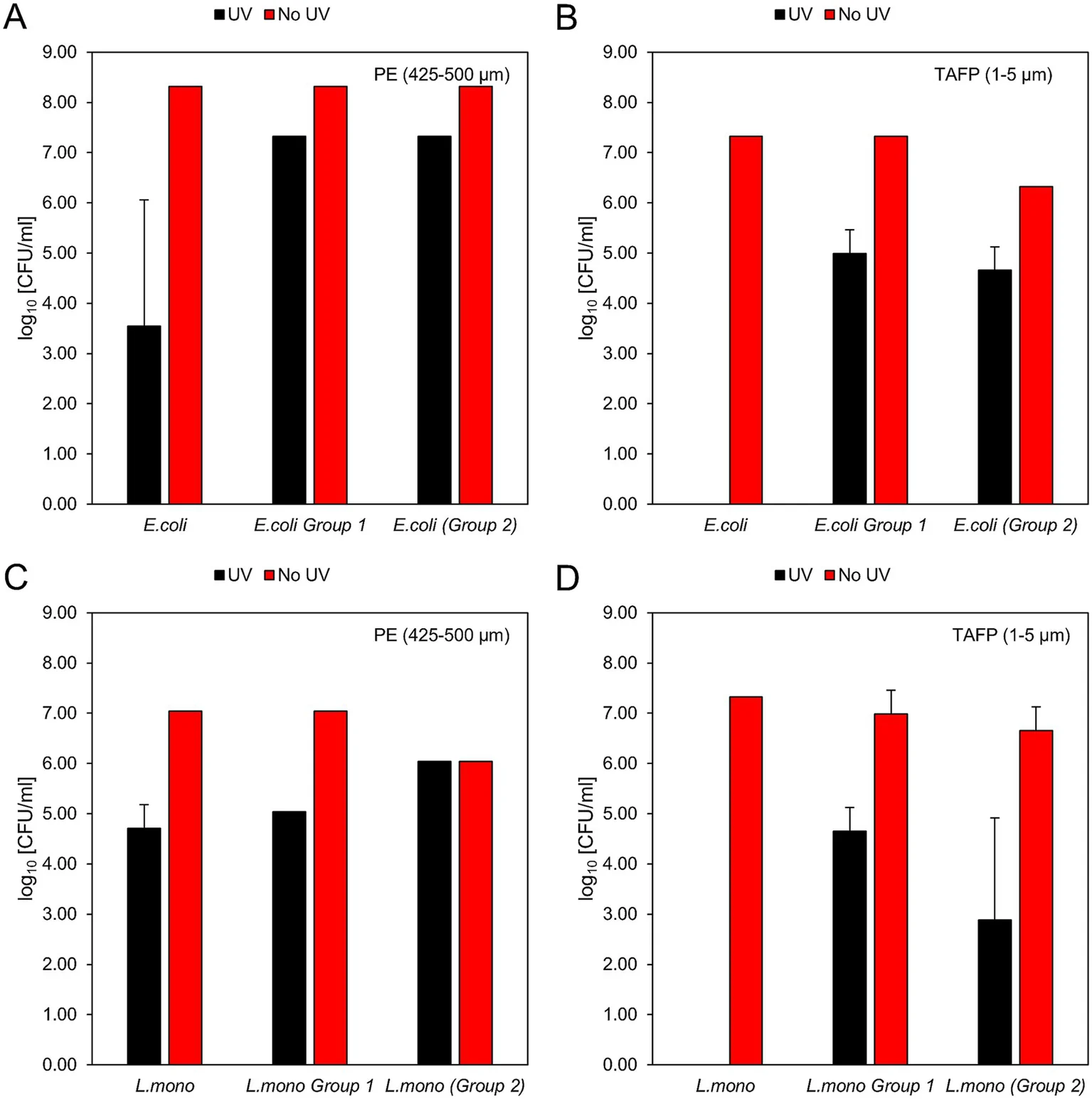
Effect of UV light on the growth of *Escherichia coli* (MG 1655) **(A** and **B)** and *Listeria monocytogenes* (EGDe) **(C** and **D)** in the presence of microplastics. Bacteria, dried on metal plates, were exposed to 1 h of UV light. *Escherichia coli* was tested alone and in the presence of MNPs. Group 1 represents *Escherichia coli* that were incubated with microplastics overnight, while Group 2 represents *Escherichia coli* that were exposed to microplastics only briefly before the UV exposure. Bars represent bacterial concentrations [log_10_(CFU/mL)] measured after 1 h of UV exposure, whereas control samples labeled “No UV” were exposed to daylight. Error bars indicate the standard deviation (SD). Sample sizes: *Escherichia coli* (*n* = 3 per condition), *Listeria monocytogenes* (*n* = 3 per condition).

In case of *E. coli*, UV treatment of samples without MNPs achieved a 4.7–7.3 log_10_ reduction, demonstrating the effectiveness of this treatment (Figures 5A,B). In contrast, presence of MNPs significantly reduced the efficacy of UV treatment. Regardless of the type of plastics, MNPs in Group 1 and Group 2 decreased the log_10_ reduction of *E. coli* by UV treatment compared to the control group, with bacterial levels being almost unaffected (Figures 5A,B; Supplementary Table 5). These results are in good accordance with previous studies demonstrating that microplastics significantly reduced the efficiency of ultraviolet and chlorine disinfection in water/wastewater treatment using *E. coli* as an indicator strain (15, 37).

The results for *L. monocytogenes* confirm these overall findings. As for *E. coli*, for the control, a 2.3–7.3 log_10_ reduction was found as well as a reduced efficacy in presence of TAFP for both Groups. Interestingly for PE, while Group 2 of *L. monocytogenes* was not inhibited at all by UV light, Group 1 and the bacterial control were reduced by 2 log_10_, similarly to the control group without PE (Figures 5C,D; Supplementary Table 6). Previous studies show that MPs and MP associated biofilms can influence the efficacy of drinking water UV disinfection processes, primarily by shielding bacteria aggregated with MPs from UV exposure (37, 38), which is the mechanism that likely explains the effects observed in this study. However, the magnitude of this shielding appears to depend on both MP type and bacterial species. Although the literature remains limited in scope, one study reported that UV inactivation in water varied by plastic and species: on PVC, multidrug-resistant *E. coli* was inactivated faster than enterococci, whereas on PE, *E. coli* showed little to no inactivation and enterococci were inactivated more slowly than on PVC (37). These results underscore that both polymer type and bacterial traits can modulate UV disinfection performance and that future studies should include a more diverse set of bacterial species, MPs and disinfection environments (e.g., drinking water and surfaces) in order to improve microbial safety management and regulatory frameworks for MP contamination.

## 4 Conclusion

The presence of micro- and nanoplastics (MNPs) has been increasing in the environment for decades and is now recognized as a major pollutant of concern in natural and urban settings, including agriculture and food production. Previously, research focused on direct health hazards and on MNPs as vectors for microbial biofilms. This study is the first to provide experimental evidence that MNPs at high concentrations directly affect the efficacy of disinfection methods routinely used in the food industry for surface cleaning. The results show that the efficacy of UV-based disinfection is reduced, whereas thermal inactivation is unaffected. For chemical disinfection, certain biocide classes are impacted, with plastic type playing a crucial role due to biocide–MNP adsorption, while species differences might also contribute. Overall, this study provides the first evidence of adverse effects on food industry disinfection methods and highlights the need for more extensive studies investigating a wider range of MNPs, including different polymer types, sizes, concentrations and shapes but also additional bacterial species (especially strong biofilm formers), biofilm state and chemical disinfectants. Furthermore, analysis of the extent of the change in disinfectant effectiveness and the mechanism behind it is important to understand potential risks in everyday conditions. To accurately assess the natural conditions, it is vital to examine and uncover the effects of microplastics in worst case scenario concentrations, but also to examine whether these concepts apply in environmentally relevant ones. Moreover, the shielding effects of MNPs towards microorganisms points out that microplastic contamination and disinfection procedure must be carefully considered, not only in the food industry, but also in other sectors with strict hygienic requirements (e.g., health care).

## Data Availability

The datasets presented in this study can be found in online repositories. The names of the repository/repositories and accession number(s) can be found at: https://doi.org/10.5281/zenodo.18641889.
