# Supplementary material for: Bacterial-microplastic interaction can affect disinfection efficiency of methods routinely used in the food industry
Source: Front Nutr. 2026 Aug 19;13:1812387. doi: 10.3389/fnut.2026.1812387 (PMC13533754; doi:10.3389/fnut.2026.1812387)
Supplement: Supplementary file 1 [file Image_1.pdf]

frontiers

Supplementary Material

1 Supplementary Figures and Tables

1.1 Supplementary Figures

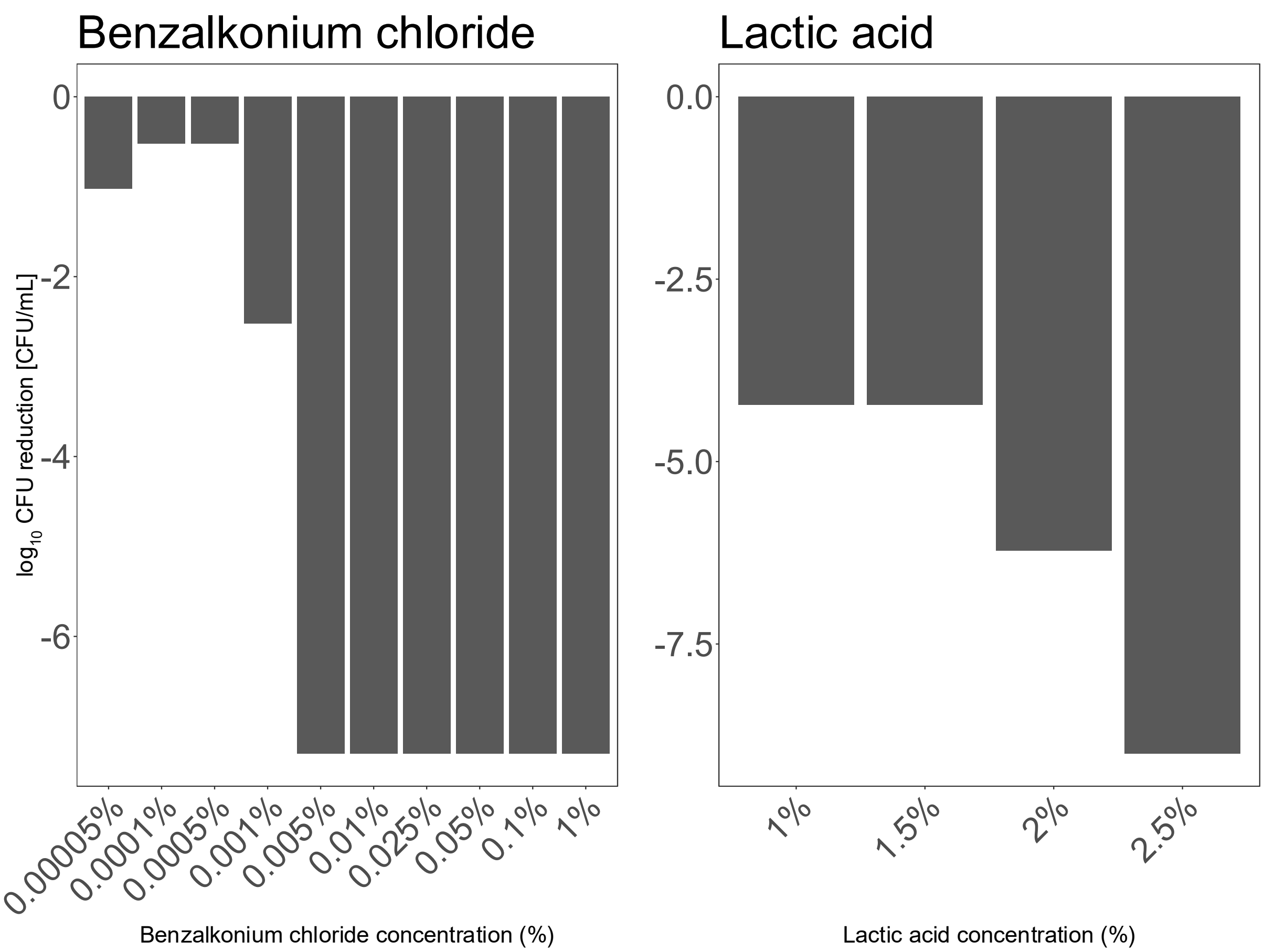

Supplementary Figure 1 Testing the disinfection efficiency of benzalkonium chloride and lactic acid at different concentrations. The chemical disinfectants were tested on E. coli (MG 1655). The bars represent the mean log_{10} CFU reduction (CFU/mL) of the bacteria after 30 minutes of exposure to the disinfectants (n=2).

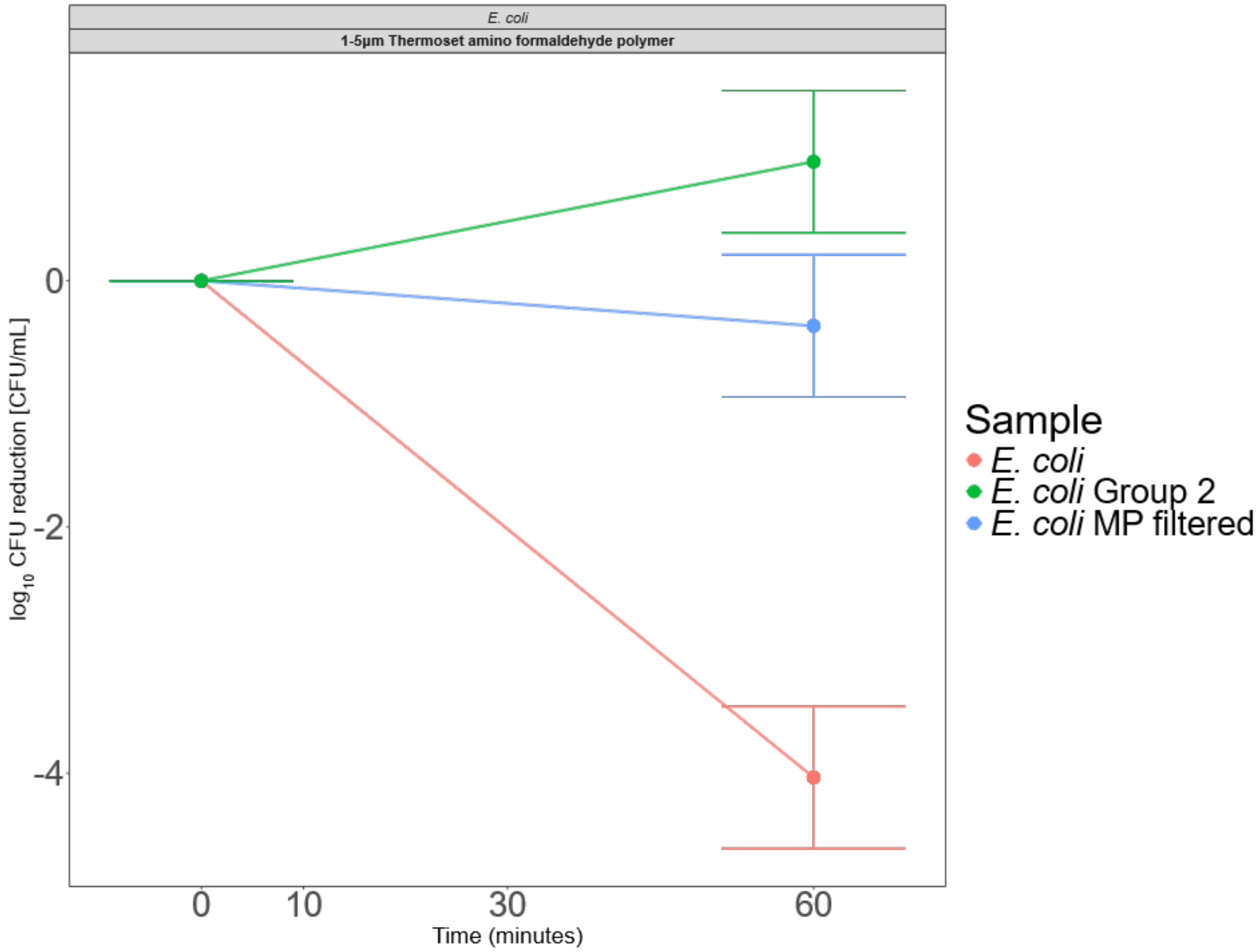

Supplementary Figure 2 Effect of TAFP exposed filtered benzalkonium chloride on the growth of E. coli (MG 1655). Benzalkonium chloride (final concentration 0.001%) was exposed to TAFP for 60 minutes and subsequently filtered to remove MNPs. Bacteria were exposed to the filtered benzalkonium chloride, and were sampled before exposure and 60 minutes (E. coli MP filtered). Other groups tested were E. coli Group 2 and E. coli with no MNP exposure. The dots represent mean $\log_{10}$ CFU reduction (CFU/mL) after subtraction of the initial bacterial concentration and the corresponding control concentration at each time point. Error bars indicate the standard deviation (SD). The experiment was performed in biological triplicates (n=3).
